# The Role of Leptin in Systemic Lupus Erythematosus: Is It Still a Mystery?

**DOI:** 10.7759/cureus.26751

**Published:** 2022-07-11

**Authors:** Nicole Villa, Omar Badla, Raman Goit, Samia E Saddik, Sarah N Dawood, Ahmad M Rabih, Ahmad Mohammed, Aishwarya Raman, Manish Uprety, Maria Jose Calero, Maria Resah B Villanueva, Narges Joshaghani, Lubna Mohammed

**Affiliations:** 1 Internal Medicine, California Institute of Behavioral Neurosciences & Psychology, Fairfield, USA; 2 General Surgery, California Institute of Behavioral Neurosciences & Psychology, Fairfield, USA; 3 Pediatrics, California Institute of Behavioral Neurosciences & Psychology, Fairfield, USA; 4 Gynecology, California Institute of Behavioral Neurosciences & Psychology, Fairfield, USA; 5 Research, California Institute of Behavioral Neurosciences & Psychology, Fairfield, USA; 6 Psychiatry and Behavioral Sciences, California Institute of Behavioral Neurosciences & Psychology, Fairfield, USA

**Keywords:** systematic review, sle, systemic lupus erythematosus, leptin receptor, leptin

## Abstract

Systemic lupus erythematosus (SLE) is a chronic inflammatory connective tissue disease with varying clinical manifestations. Recent studies have proposed that leptin may be related to SLE development. This study aims to assess current information regarding the relationship between leptin and SLE. A systematic search was done using PubMed, Google Scholar, ScienceDirect, Epistemonikos, and Cochrane Library databases. Studies published in the English language in the last 10 years were selected based on predefined eligibility criteria. The quality of the studies was evaluated using the Newcastle-Ottawa Scale and the Assessment of Multiple Systematic Reviews 2 tool.

A total of 12 studies were included in this systematic review. These included systematic reviews/meta-analyses, cross-sectional studies, and case-control studies*.* Based on the findings of this review, we conclude thatleptin is significantly elevated in SLE patients; however, it does not seem to correlate with disease activity. The exact mechanism of leptin in the pathogenesis of the disease remains unknown and further research is needed regarding this aspect.

## Introduction and background

Systemic lupus erythematosus (SLE) is a chronic autoimmune disease with varying clinical manifestations. This inflammatory connective tissue disorder affects more females than males [1]. Although it can present at any age, women of childbearing age are at a higher risk [2]. In the United States, the overall incidence of SLE from 2002 to 2009 was estimated to be 5.1 per 100,000 person-years. This data indicated that in 2018 approximately 14,263 people were newly diagnosed with this disease. Concerning race, in both male and female groups, the incidence was the highest among African Americans [3]. The etiology and pathogenesis of SLE are not clearly understood, but it is believed that it can be the result of a complex interaction of several factors, such as hormones, genetic predisposition, immune dysfunction, environment, and ethnicity [4,5]. SLE is characterized by the breakdown of self-tolerance, which leads to the production of autoantibodies, the deposition of immune complexes in several organs, and the presence of high levels of proinflammatory cytokines in the serum [5,6]. In patients with lupus, there is an increase in mortality due to infection, malignancy, atherosclerosis, and renal disease, which emphasizes the importance of understanding this disorder [2].

The adipose tissue is an endocrine organ that secretes adipokines such as leptin, adiponectin, resistin, and visfatin. Leptin is known to have a similar function to inflammatory cytokines including interleukin (IL)-1, IL-6, and tumor necrosis factor-alpha (TNF-α) [7]. This gives leptin a role that goes beyond its well-known function of appetite regulation, introducing it as an important player in the immune system. In human beings, leptin has been associated with chronic inflammatory conditions such as rheumatoid arthritis, SLE, and intestinal inflammatory disease, among other conditions. High levels of leptin are associated with a decrease in T-regulatory cells (TReg) and an increase in Th17 cells, a process seen in the development of SLE [7,8]. Polymorphisms in genes that encode for leptin receptors have also been studied, and there is evidence that certain alleles are associated with either a higher or lower risk of SLE in different ancestral groups [9]. In mouse models of lupus, it has been shown that leptin promotes spontaneous lupus and that its antagonism decreases disease manifestations and aids in immune regulation [10].

In recent years, several studies have described a relationship between leptin levels and SLE, but the results have varied widely [11]. In this systematic review, we aim to assess current literature on the topic to further investigate this association. We hope to provide a better understanding of the available evidence and help determine its clinical significance.

## Review

Methods

This systematic review was conducted based on the Preferred Reporting Items for Systematic Reviews and Meta-Analyses (PRISMA) 2020 guidelines [12].

Search Strategy

We thoroughly searched PubMed, Cochrane Library, Google Scholar, ScienceDirect, and Epistemonikos. We used appropriate keywords and Medical Subject Heading (MeSH) terms to identify all potentially relevant articles describing the relationship between leptin and SLE. The keywords used included leptin, leptin receptor, level, systemic lupus erythematosus, and SLE. We applied the Boolean method to combine the keywords and MeSH terms to synthesize a uniform search through the various databases. The last date of the search through all the databases was April 10th, 2022. The description of the terms used in each database can be seen in Table 1.

**Table 1:** Terms used for the bibliographic search with their corresponding filters. SLE: systemic lupus erythematosus

Database	Keywords	Search strategy	Filters
PubMed	Leptin, Leptin receptor, level, systemic lupus erythematosus, SLE	Systemic Lupus Erythematosus OR SLE OR (“Lupus Erythematosus, Systemic/blood”[Majr] OR “Lupus Erythematosus, Systemic/genetics”[Majr] OR “Lupus Erythematosus, Systemic/immunology”[Majr] OR “Lupus Erythematosus, Systemic/metabolism”[Majr] OR “Lupus Erythematosus, Systemic/pathology”[Majr] OR “Lupus Erythematosus, Systemic/physiology”[Majr] OR “Lupus Erythematosus, Systemic/physiopathology”[Majr] ) AND Leptin OR (“Leptin/blood”[Majr] OR “Leptin/genetics”[Majr] OR “Leptin/immunology”[Majr] OR “Leptin/metabolism”[Majr] OR “Leptin/physiology”[Majr] ) AND Leptin receptor OR (“Receptors, Leptin/blood”[Majr] OR “Receptors, Leptin/chemistry”[Majr] OR “Receptors, Leptin/genetics”[Majr] OR “Receptors, Leptin/immunology”[Majr] OR “Receptors, Leptin/metabolism”[Majr])	Last 10 years, free full text, English language, humans
ScienceDirect	Leptin, systemic lupus erythematosus, SLE	Leptin AND SLE OR Systemic Lupus Erythematosus	2012–2022 research articles, review articles
Cochrane	Leptin, level, systemic lupus erythematosus, SLE	SLE AND Leptin	2012–2022
Epistemonikos	Leptin, systemic lupus erythematosus, SLE	SLE OR Systemic Lupus Erythematosus AND Leptin	Last 10 years
Google Scholar	Leptin, level, systemic lupus erythematosus	Systemic Lupus Erythematosus AND Leptin Levels	2012–2022

Eligibility Criteria

We restricted our search to online records in the English language, available as free full texts, and issued in the last 10 years (April 2012 to April 2022). We only included studies conducted among human participants. We restricted our choice of studies to systematic reviews, meta-analyses, cohort studies, case-control studies, and cross-sectional studies that provided data regarding plasma/serum leptin levels. We excluded case reports, editorials, animal studies, and gray literature.

Risk of Bias in Individual Studies

We assessed each study for the potential risk of bias. We evaluated case-control studies using the Newcastle-Ottawa Scale (NOS), and for cross-sectional studies, we used an adapted version of the NOS. Systematic reviews and meta-analyses were assessed using the Assessment of Multiple Systematic Reviews 2 (AMSTAR 2) tool. Two authors (Nicole Villa and Omar Badla) independently reviewed the quality of the studies, and a score of at least 70% for each assessment tool was accepted.

Results

**Figure 1:**
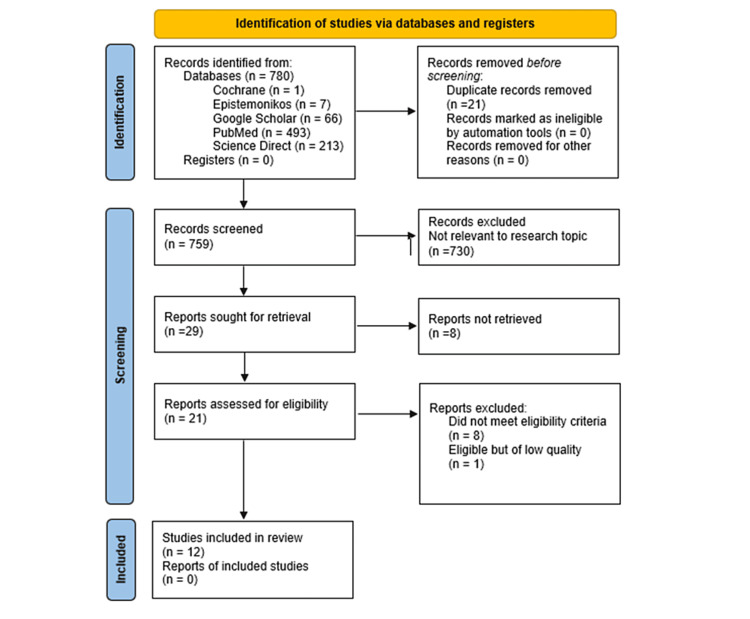
PRISMA flowchart of the study search selection. PRISMA: Preferred Reporting Items for Systematic Reviews and Meta-Analyses

We identified a total of 780 records using the various search strategies across the databases. Out of the 780 records, 493 originated from PubMed, 66 from Google Scholar, 213 from ScienceDirect, seven from Epistemonikos, and one from the Cochrane Library. No other resource was used. We thoroughly checked all our records, organized them in alphabetical order using endnote, and then manually excluded 21 duplicates. The remaining 759 records were thoroughly screened for relevance based on titles and abstracts, after which 730 records were excluded. Hence, 29 articles were sought for retrieval, and after checking free full texts, we further removed eight records. We assessed 21 reports for eligibility, of which eight articles did not fulfill the inclusion and exclusion criteria. There was one article that met the study’s criteria but was of low quality and was excluded. Therefore, we included 12 articles, which included systematic reviews/meta-analyses, cross-sectional studies, and case-control studies. The complete PRISMA flowchart of our review is shown in Figure 1.

Study Characteristics

The main characteristics of the studies included in this systematic review are shown chronologically in Table 2. All the included studies were journal articles, of which six were case-control studies, four were cross-sectional studies, and two were systematic reviews/meta-analyses.

**Table 2:** The main characteristics of case-control and cross-sectional studies included in the review. *: In the study by Balaji et al., the exact number of female and male participants was not mentioned but the article did report a male:female ratio of 1:12 in cases as well as in controls. N/A: not applicable; F/M: female/male; SLE: systemic lupus erythematosus; SLICC: Systemic Lupus International Collaborating Clinics; ACR: American College of Rheumatology; ELISA: enzyme-linked immunosorbent assay

Author	Year	Region	Study Design	Case		Control		Criteria of the classification of SLE	Measurement of leptin
				n	Sex ratio (F/M)	n	Sex ratio (F/M)		
Liangliang et al. [13]	2015	China	Case-control	87	76/11	85	71/14	SLICC	ELISA
De Souza Barbosa et al. [14]	2015	Brazil	Case-control	52	52/0	33	33/0	ACR	ELISA
Margiotta et al. [15]	2016	Italy	Case-control	13	13/0	11	11/0	SLICC	ELISA
Hong Miao et al. [16]	2017	China	Case-control	633	573/60	559	553/6	ACR	ELISA
Wang et al. [17]	2017	China	Case-control	47	47/0	25	25/0	ACR	ELISA
Mohammed et al. [18]	2017	Egypt	Case-control	40	40/0	20	20/0	ACR	ELISA
Muhammad et al. [19]	2019	Indonesia	Cross-sectional	35	35/0	35	35/0	Not reported	ELISA
Balaji et al. [20]	2020	India	Cross-sectional	80	N/A*	40	N/A*	SLICC	ELISA
Aghdashi et al. [21]	2020	Iran	Cross-sectional	40	40/0	40	40/0	ACR	ELISA
Kondratyeva et al. [22]	2021	Russia	Cross-sectional	46	46/0	N/A	N/A	ACR	ELISA

Because of the few systematic reviews and meta-analyses published in the field, we elected to include them in this review. The characteristics of these studies are shown in Table 3.

**Table 3:** The main characteristics of the systematic review and meta-analysis included in the review. SLE: systemic lupus erythematosus; OR: odds ratio; CI: confidence interval

Author	Year	Type of study	Databases reviewed	Number of studies included	Total participants	Inclusion and exclusion criteria	Outcomes and key points	Funding
Lee et al. [23]	2018	Meta-analysis	Medline, Embase, Cochrane	20	1,333 patients and 1,048 controls	Inclusion criteria: case-control, cross-sectional, or cohort studies with data on serum/plasma leptin levels in case and control groups. No language and race restrictions. Exclusion criteria: overlapping or insufficient data, reviews, and case reports	Leptin levels were elevated in SLE patients regardless of ethnicity, data type, sample size, and matched variables. The study proposed that leptin may have an important role in the pathogenesis of SLE, but the exact mechanism is not clear	No external funding
Yuan et al. [4]	2020	Systematic review and meta-analysis	PubMed, Embase, Cochrane, China National Knowledge Infrastructure, China WanFang, China Weipu	34	1,744 patients and 1,411 controls	Inclusion criteria: cohort, case-control, and cross-sectional studies with data on the serum/plasma leptin levels, leptin receptor levels, and relevant gene polymorphism in case and control groups, studies with predefined SLE criteria and measurement methods, studies with OR estimates with 95% CI and p-values. Exclusion criteria: case reports, editorials, letters, comments, review articles, meta-analysis, and non-human subjects	Leptin levels were higher in SLE patients regardless of source, sample size, or assay method. In terms of ethnicity, leptin levels were elevated in Asian, African, North American, and European groups. SLE patients showed a decreased trend of leptin receptor levels	Received external funding

Discussion

In recent years, the relationship between SLE and leptin levels has received more attention, and several studies have been performed on this topic. However, the results vary widely making it challenging to draw solid conclusions from a single study. Due to the importance of SLE, in this systematic review, we analyzed recent literature to help elucidate the role of leptin in this autoimmune condition.

The Possible Role of Leptin in the Pathogenesis of SLE

It has been described that leptin plays a role in immunity, regulating both the innate and adaptative responses. In the adaptative response, leptin decreases the proliferation of TReg cells while it promotes the proliferation of naïve T and B cells. In the innate response, leptin increases the cytotoxicity of natural killer cells and enhances the chemotaxis of macrophages and monocytes. Leptin also increases the expression of B-cell lymphoma 2 (BCL-2) in T cells, delaying their apoptosis [23,24]. In mouse models, it was shown that leptin-deficient mice had a higher number and activity of TReg cells which provided them resistance to autoimmune diseases [25].

Leptin favors a switch toward Th1 inflammatory cytokines such as interferon-gamma, IL-6, and TNF-α. On the other hand, it downregulates the Th2 anti-inflammatory cytokines including IL-4 and IL-10. In doing so, leptin creates an imbalance between Th1 and Th2 lymphocytes, and at the same time, it facilitates Th17 differentiation. Leptin has been shown to modulate the development of B lymphocytes and activate them to secrete cytokines, worsening the inflammatory state. A deficit in TReg cell function and a notable Th17 differentiation are also observed in lupus [4,24]. Therefore, by the mechanisms described, leptin may be involved in the pathogenesis of lupus.

Leptin Levels in Patients With SLE

In all the case-control and cross-sectional studies included in this review, leptin was measured by enzyme-linked immunosorbent assay (ELISA). We found that most of the studies showed that SLE patients had higher leptin levels in plasma or serum in comparison to healthy controls. In a cross-sectional study that included only SLE patients, Kondratyeva et al. reported that 76% of the patients had hyperleptinemia [22]. In contrast to these findings, in a study performed on an Indonesian population, the SLE group had a higher mean leptin level, but the difference was not statistically significant [19]. Another study in which no difference was seen between the two groups was a case-control study performed on an Italian population by Margiotta et al. [15].

However, in the systematic review and meta-analysis included in this review, it was shown that leptin was significantly higher in SLE patients. Lee et al. reported in their meta-analysis that leptin levels were higher in patients with SLE independent of sex, body mass index, sample size, age, or data type [23]. Whereas the subgroup analysis by sex reported by Yuan et al. showed that leptin was significantly elevated in the female group with SLE but not in the male group [4]. This finding could be explained by the fact that in many of the studies that they included, the population was mainly female, which is understandable because lupus is much more prevalent in females [4,26].

Concerning the relationship between ethnicity and leptin levels in SLE patients, the study by Yuan et al. included Asian, North American, European, South American, and African participants, and the study by Lee et al. included Caucasian, Asian, Arab, Latin American, and mixed ethnic populations. Both studies showed that leptin was significantly higher in SLE patients belonging to the Asian and European groups [4,23].

Disease Activity and Leptin

The Systemic Lupus Erythematosus Disease Activity Index (SLEDAI) is a tool designed to help clinicians measure disease activity in patients with lupus. The index was developed by rheumatologists with expertise in SLE, and since its introduction, it has been widely used in clinical practice and in several studies [27]. This index takes into consideration clinical manifestations and laboratory values and aims to reflect the disease activity within the last 10 days. Based on the score, clinicians can determine if the patient has an active or inactive disease, the severity of the active disease, and whether there has been a flare-up [28].

Of the 12 studies included in the review, only 10 had information regarding the relationship between leptin levels and disease activity measured by SLEDAI. Out of these, only three studies showed a positive correlation between disease activity measured by SLEDAI and leptin level [17,21,22]. In 2020, Yuan et al. showed that even though there was a trend toward higher leptin levels in patients with a SLEDAI score above 10, this was not significant when compared to those with a score lower than 10 [4].

It is not clear whether leptin is related to specific clinical manifestations. Mohammed et al. showed that leptin was higher in patients who had alopecia and renal affection [18]. On the other hand, Balaji et al. described that leptin was higher in patients with malar or discoid rash, alopecia, and those with musculoskeletal manifestations [20]. However, this data was not significant, and to our knowledge, to date, there are not many studies that show that leptin is higher or lower with specific clinical manifestations.

LEP and Leptin Receptor Gene Polymorphisms in SLE

Recent studies have analyzed the association of leptin-related polymorphisms in patients with lupus to decipher its possible impact on susceptibility to the disease.

In 2016, Li et al. conducted a study in a Chinese population in which they investigated the LEP and leptin receptor (LEPR) gene single-nucleotide polymorphisms in patients with SLE and controls. They did not find an association between the different polymorphisms and the risk for SLE. However, in the LEP gene, they showed that lupus patients with pericarditis had higher frequencies of the TT genotype and T allele frequencies of the rs2071045 polymorphism. Concerning the LEPR, they found an association between the GA/GG genotype and G allele frequencies of the rs3806318 polymorphism and the presence of photosensitivity. As to the SLEDAI score, there was no difference in genotype distribution in patients with a score above 10 when compared to those with a score below this value [16].

Zhao et al. performed a large study with a total of 15,706 participants from different ancestral groups and reported similar results. The investigators found that in LEP the A allele of rs3828942 increased the risk of SLE in African Americans. Conversely, the A allele of rs12706832 was related to a decreased risk in the same population. LEPR rs1892535 and rs6690625 were associated with a diminished risk of lupus in a population Hispanic enriched for the Amerindian European admixture. However, a consistent association between the different polymorphisms in multiple ancestral groups was not found. Therefore, the leptin-related polymorphisms analyzed were not associated with a higher susceptibility to developing lupus [29].

Body Mass Index and Leptin

Because leptin is related to adipose tissue, body mass index (BMI) is a factor that should be taken into consideration due to the effect it can have on leptin levels. Paul et al. described that in healthy subjects, serum leptin concentration progressively augmented with an increase in BMI [30]. This association was also reported by Martins et al.; in their study, BMI was not only strongly associated with leptin but was also considered a great predictor of hyperleptinemia on receiver operating characteristic (ROC) analysis [31].

Mohammed et al. reported similar findings; in their study, the mean BMI in SLE patients was 25.36 kg/m^2^, and it had a positive correlation with leptin levels. They also described an association between leptin and total cholesterol values [18]. Barbosa et al. reported that SLE subjects had a higher BMI in comparison to healthy controls, and BMI had a positive correlation with leptin levels only in lupus patients. However, in their study, leptin was associated with high-density lipoprotein (HDL) cholesterol and not with total cholesterol [14]. These findings go hand in hand with those of Balaji et al. who also reported that leptin was correlated with BMI and HDL cholesterol [20]. In our systematic review, each study had different inclusion and exclusion criteria; in some cases, just accepting patients within a specific BMI range which could be related to their findings on leptin levels.

Limitations

This study has some limitations that should be considered. First, this review only considered studies that were published in the last 10 years, published in the English language, and that were available as free full text. Second, most of the studies included had small sample sizes, and the patients had different clinical features, demographic characteristics, and received different treatments. The variables controlled differed widely making it challenging to compare the data reported by different studies. Nevertheless, our study also has its strengths. In comparison with a single study, in this systematic review, we were able to provide more knowledge about the existing relationship between leptin and SLE. We consider that the information provided in this review can guide future studies on this topic.

## Conclusions

Leptin is significantly elevated in SLE patients, but the exact mechanism of how it is related to the pathogenesis of the disease remains unclear. Based on the findings of this review, leptin levels do not appear to correlate with disease activity in lupus patients. The distinct gene polymorphisms of LEP and LEPR could be related to certain clinical manifestations, but they do not appear to increase the risk for the development of SLE. Due to the association between BMI and leptin levels, BMI is a variable that should be taken into consideration when evaluating leptin in SLE patients.

Most of the studies on this topic were performed on a small number of patients and had very different inclusion and exclusion criteria, which could explain the controversial results obtained. Further research is needed to explore the connection between leptin and lupus, to clarify its role in the development of the disease, and whether it can be used as a tool to aid early diagnosis. For future investigations on the topic, we suggest conducting a study with a larger sample of patients, and when examining leptin levels in SLE patients, taking into consideration variables such as sex, ethnicity, treatment received, and BMI.
